# Estimation of the strength of mate preference from mated pairs observed in the wild

**DOI:** 10.1111/evo.14397

**Published:** 2021-12-02

**Authors:** Erin Clancey, Timothy R. Johnson, Luke J. Harmon, Paul A. Hohenlohe

**Affiliations:** ^1^ Department of Mathematics and Statistical Science University of Idaho Moscow Idaho 83844 USA; ^2^ Department of Biological Sciences Institute for Bioinformatics and Evolutionary Studies, University of Idaho Moscow Idaho 83844 USA

**Keywords:** Assortative mating, mate preference, maximum likelihood, quantitative genetics models, sexual selection

## Abstract

A number of key processes in evolution are driven by individuals preferring mates with particular phenotypes. However, despite long‐standing interest, it is difficult to quantify the strength of mate preference from phenotypic observations in nature in a way that connects directly to key parameters in theoretical models. To bridge the gap between mathematical models and empirical data, we develop a novel maximum likelihood‐based method to estimate the strength and form of mate preference, where preference depends on traits expressed in both males and females. Using simulated data, we demonstrate that our method accurately infers model parameters, including the strength of mate preference and the optimal offset match between trait values in mated pairs when model assumptions are satisfied. Applying our method to two previous studies of assortative mating in marine gastropods and the European common frog, we support previous findings, but also give additional insight into the role of mate preference in each system. Our method can be generalized to a variety of plant and animal taxa that exhibit mating preferences to facilitate the testing of evolutionary hypotheses and link empirical data to theoretical models of assortative mating, sexual selection, and speciation.

In many species, individuals prefer and choose mates with particular characteristics. Mate preference, the propensity for individuals to select mates on the basis of phenotypes, is a pervasive, highly complex, and very important driver of evolution. For instance, mate preferences can generate assortative mating, produce persistent selection on mating success (Lande 1981; Kirkpatrick and Ravigné 2002), and lead to speciation even in the face of gene flow (Dieckmann and Doebeli 1999; Kirkpatrick and Ravigné 2002; Pinho and Hey 2010). In general, there are two predominant types of mate preference rules that can give rise to assortative mating but may otherwise produce different evolutionary outcomes: (i) preference for a mate with a certain trait value regardless of the chooser's own trait value (preference/trait), and (ii) preference for a mate on the basis of a match with their own phenotype (matching) (Kopp et al. 2018). Although both types of mate preference rules can generate sexual selection and have important evolutionary consequences, matching rules are more commonly assumed in theoretical models of sympatric speciation and divergence with gene flow than preference/trait rules (see Kopp et al. 2018). Matching mate preferences can decrease gene flow between sympatric or parapatric groups that are phenotypically divergent (Doebeli and Dieckmann 2000; Servedio 2004; Otto et al. 2008). Conversely, trait matching preferences can also act as a strong force of stabilizing sexual selection that stymies divergence (Kirkpatrick and Nuismer 2004; Servedio and Kopp 2012), or these preferences can lead to the evolution of sexual dimorphism instead of diversification (Bolnick and Doebeli 2003). The evolution of preferences themselves may also be idiosyncratic or system‐specific. For example, *Heliconius* butterflies have mimetic color patterns that are under frequency‐dependent selection (Chamberlain et al. 2009), and mate preferences evolve adaptively to produce the most fit offspring (see Jiang et al. 2013). Similarly, mate preference for a phenotypically or genetically similar mate may evolve due to outbreeding depression (Epinat and Lenormand 2009) and may contribute to population structure in small populations that may be of conservation interest (e.g., Langin et al. 2015). Henceforth, we consider the specific case of the trait matching rule and define mate preference as the preference for a mate conditioned upon the individual chooser's own phenotype, which implies that phenotypic traits can be measured in both sexes.

Mate preference is fundamental to biological questions linking ecology, phenotypic evolution, and the genetic basis for mating traits, yet our capacity to measure mate preference and study its consequences in natural systems is highly limited. Much of what we know and the predictions we are able to make about evolutionary processes involving mate preference stem from theoretical models, laboratory studies, and simulations, with less emphasis on field studies (Turelli et al. 2001; Kirkpatrick and Ravigné 2002; Gavrilets 2004; Carvajal‐Rodriguez and Rolán‐Alvarez 2014). This is because in most, if not all cases, it is impossible to directly measure mate preference in the wild. Experimentally, mate preferences can be studied using mate‐choice trials, but such trials are accompanied by assumptions and confounding effects not found in nature and are not feasible in many study organisms (Johnson and Marzluff 1990; Dougherty 2020). Simulation studies connect a mathematical framework to the sampling process and allow us to study the effects of specific parameters on evolutionary outcomes given a set of assumptions. However, mathematical models go notably untested with field data, leaving a lacuna between theory and the natural world. One tractable approach in studies of natural populations is to infer mate preference via a Pearson correlation coefficient calculated between quantitative traits measured within known mated pairs (Jiang et al. 2013). This is valuable because it has allowed us to observe presence or absence of assortative mating across a variety of taxa (de Cara et al. 2008; Jiang et al. 2013). A correlation can be used as evidence that assortative mating may be occurring in a population, however, it cannot accurately measure the strength of mate preference directly, nor can correlations help distinguish between mate preference rules. Furthermore, correlation coefficients do not appear as parameters in analytical or simulation‐based models of sexual selection or divergence with gene flow (de Cara et al. 2008). To provide a link between theory and data, the strength of mate preference must be directly estimated as a parameter value.

Here we develop a likelihood‐based method adapted from the mathematical framework given in Kirkpatrick and Nuismer (2004) to infer the strength of mate preference from observational data collected in the wild. Our method requires observations of phenotypes of males and females in mated pairs, as well as phenotypes of randomly sampled individuals (although not strictly required in all cases, see the “Likelihood Function” section) of reproductive capacity in a population. We know of one other family of methods to estimate mate preference from similar data (Rolán‐Alvarez et al. 2015; Fernández‐Meirama et al. 2017; Estévez et al. 2018). These approaches are based on fitting a strength parameter to indices of assortative mating for each pair that are scaled to approximate probabilities. We take a more direct statistical approach by estimating all parameters via maximum likelihood, which also provides for the calculation of standard errors or profile likelihood functions to gauge the (im)precision in the estimation, as well as formal inferences such as statistical tests and confidence intervals. This approach is also easily extended to generalizations of the model. We also include a parameter that allows for offset matching, where the phenotype of the most preferred partner differs from the chooser's own phenotype by a fixed value instead of strict trait matching only, as is common in most mating functions (e.g., Lande and Arnold 1983; Arnqvist et al. 1996; Dieckmann and Doebeli 1999; Thibert‐Plante and Hendry 2009). In the next sections, we (i) present the mathematical framework from which we build a likelihood‐based method to estimate parameter values. Using the mathematical framework in (i), we (ii) demonstrate why using a correlation to measure the strength of mate preference is insufficient and can generate spurious results. Then, we (iii) evaluate the performance of the maximum likelihood estimates with simulated data, and (iv) analyze and interpret data from two previously published studies on assortative mating using our methodology, which we have made publicly available in an package called matepref (Clancey and Johnson 2021) for R (R Core Team 2021).

## The Statistical Model

### MATE PREFERENCE MODEL

Our model is designed to estimate the strength of mate preference when individuals prefer a phenotypically similar mate (i.e., trait matching) or a mate with a larger or smaller trait value relative to themselves (i.e., offset matching) on a continuous scale. We typically rely on having a single quantitative trait that can be measured in both sexes. In some cases it may also be appropriate to have two quantitative traits, one that is measured in males and one that is measured in females, where each trait is important in the context of mating. Specifically, our model is a statistical application of a theoretical model proposed by Kirkpatrick and Nuismer (2004), which we adapt for the purpose of estimating parameters pertaining to mate preference from observations of trait values in mated pairs of organisms in the wild.

We assume individuals can move freely in space and come into contact with potential mates at random. In dioecious species, male and female traits, or in hermaphroditic species, traits of donors and recipients, hereafter X and Y, are assumed to be independently sampled from a homogeneous population. To give a general formulation of the model, we can decompose the joint distribution of traits in (X,Y) *mated pairs* of organisms into the unspecified marginal density functions $f_{X}\left(x\right)$ and $f_{Y}\left(y\right)$, a mating function describing the probability of mating between individuals with X and Y trait values, and a normalizing constant. Assuming observations within X, Y, and (X,Y) *mated pairs* are independent, the joint distribution of X and Y trait values given that the organisms are in a mated pair can be written using Bayes' theorem as

(1) $$M\left(x,y|S=1\right)=\frac{f_{X}\left(x\right)f_{Y}\left(y\right)P\left(S=1|x,y\right)}{P(S=1)},$$

where S denotes an indicator variable for a successfully mated pair and the denominator, P(S=1), is the marginal probability of mating. The mating function we propose builds upon a Gaussian mating function from Lande (1983) and Kirkpatrick and Nuismer (2004), but we include additional parameters such that

(2) $$P\left(S=1|x,y\right)=\gamma e^{-\alpha {\left(x-y-\delta \right)}^{2}},$$

where α≥0 is a parameter describing the strength of mate preference as a function of the difference between the trait values, δ is the value of x−y that maximizes the mating probability, and γ is a parameter that equals the probability of mating when x−y=δ (i.e., the probability of mating with the perfect matching or offset matching of trait values). Now we assume the distributions $f_{X}\left(x\right)$ and $f_{Y}\left(y\right)$ to be normal density functions, or approximately normal, such that $X\overset{iid}{∼}N\left(\mu_{x},\sigma_{x}\right)$ and $Y\overset{iid}{∼}N\left(\mu_{y},\sigma_{y}\right)$. These distributions, combined with the mating function in equation (2), give us the fully specified conditional distribution of mated pairs as

(3) $$M\left(x,y|S=1\right)=\frac{f\left(x;\mu_{x},\sigma_{x}^{2}\right)f\left(y;\mu_{y},\sigma_{y}^{2}\right)e^{-\alpha {\left(x-y-\delta \right)}^{2}}}{\;\int \int \;\;f\left(x;\mu_{x},\sigma_{x}^{2}\right)f\left(y;\mu_{y},\sigma_{y}^{2}\right)e^{-\alpha {\left(x-y-\delta \right)}^{2}}\;dx\;dy}.$$

Note this distribution is not dependent on the parameter γ. The denominator of equation (3) is equal to the marginal probability of mating and for computational purposes can be written in closed‐form. The double integral here is equal to the expectation

(4) $$E\left[e^{-\alpha {\left(X-Y-\delta \right)}^{2}}\right]=E\left[e^{tZ^{2}}\right],$$

where $t=-\alpha (\sigma_{x}^{2}+\sigma_{y}^{2})$ and $Z=\left(X-Y-\delta \right)/\sqrt{\sigma_{x}^{2}+\sigma_{y}^{2}}$. Because Z is a normal random variable with mean $\left(\mu_{x}-\mu_{y}-\delta \right)/\sqrt{\sigma_{x}^{2}+\sigma_{y}^{2}}$ and unit variance, $Z^{2}$ has a noncentral $\chi^{2}$ distribution with one degree of freedom and noncentrality parameter $\lambda ={\left(\mu_{x}-\mu_{y}-\delta \right)}^{2}/\left(\sigma_{x}^{2}+\sigma_{y}^{2}\right)$, and thus equation (4) is the moment‐generating function of a noncentral $\chi^{2}$ distribution. This can be written as

(5) $$E\left[e^{tZ^{2}}\right]=\frac{e^{\lambda t/(1-2t)}}{\sqrt{1-2t}},$$

(see Johnson et al. 1995) giving a fully specified statistical model to formulate the likelihood function in the following section.

### LIKELIHOOD FUNCTION

We now consider the problem of the estimation of the parameters of equation (3) with empirical data. We envision a scenario where field biologists are able to randomly sample individuals of reproductive capacity to measure a quantitative trait of interest and observe mated pairs in their study population. The data then consist of paired observations of trait values within mated pairs, $\left(x_{i},y_{i}\right)$, and additional unpaired observations of trait values of females ($x_{i}$) and males $\left(y_{i}\right)$ that are not observed as part of a mated pair. Assuming mutual independence across paired and unpaired observations, from equations (3) and (5) we have the likelihood function

(6) $$\begin{array}{ccc} L(\theta ) & = & \prod_{i\in S_{p}}\frac{f\left(x_{i};\mu_{x},\sigma_{x}^{2}\right)f\left(y_{i};\mu_{y},\sigma_{y}^{2}\right)e^{-\alpha {\left(x_{i}-y_{i}-\delta \right)}^{2}}}{e^{\lambda t/(1-2t)}/\sqrt{1-2t}}\\ & & \times \prod_{i\in S_{x}}f\left(x_{i};\mu_{x},\sigma_{x}^{2}\right)\times \prod_{i\in S_{y}}f\left(y_{i};\mu_{y},\sigma_{y}^{2}\right), \end{array}$$

where $\theta ={\left(\alpha ,\delta ,\mu_{x},\mu_{y},\sigma_{x},\sigma_{y}\right)}^{\prime }$, $S_{p}$ is the set of indices of ordered (x,y) mated pairs, and $S_{x}$ and $S_{y}$ are the sets of indices of observations x and y, respectively, that are not observed as members of a mated pair. The maximum likelihood estimate of θ can be obtained numerically by the maximization of equation (6) with respect to θ. Either or both $S_{x}$ and $S_{y}$ can be empty, in which case the corresponding terms are omitted from the likelihood function. It should be noted that $\mu_{x}$, $\mu_{y}$, and δ are not identified if $S_{x}$ and/or $S_{y}$ are empty, so offset matching cannot be distinguished from differences between the mean trait values in mated pairs. To unambiguously estimate δ it is necessary to have samples of mated pairs as well as samples of males and females that are not observed as members of mated pairs. However, if δ can be assumed to be zero, as is sometimes the case, then it can be fixed rather than estimated, and then the model is identified even with using only a sample of mated pairs.

### INTERPRETING THE MATING FUNCTION

**Figure 1:**
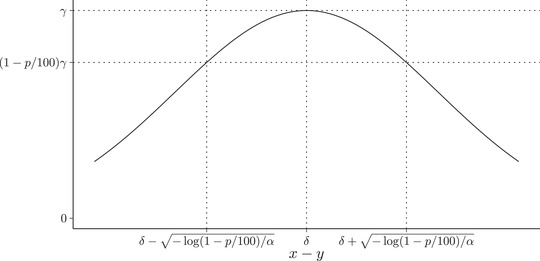
Mating probability as a function of trait differences (x−y), α, δ, and γ. The maximum probability of mating (γ) occurs when x−y=δ. A reduction of mating probability of p% can be visualized as the interval around this point.

To interpret an estimated value of α as the strength of mate preference, it is helpful to understand the mating function. The mating function in equation (2) describes the probability of mating as a function of trait differences and the parameters α, δ, and γ. It is very unlikely that the probability of mating is equal to one even in a perfectly matched pair. Therefore, we use the parameter γ as the maximum probability of mating when x−y=δ. Figure 1 depicts how the mating function depends on the trait differences x−y and the parameters α, δ, and γ. The parameter γ can be viewed as the composite effect of all factors independent of x and y on the probability of mating such as other independent traits conferring a mating advantage, availability of resources, or territories that may affect the probability of mating, or other factors that are system specific. To facilitate the interpretation of α, we can use the mating function to express an interval around δ in terms of x−y where the probability of mating is within p% of the maximum probability of mating of γ. For any p% we can define the values of x−y such that P(S=1|x,y)≥(1−p/100)γ. The interval $\delta \pm \sqrt{-\log(1-p/100)/\alpha }$ defines trait differences x−y leading to a mating probability that is within p% of the maximum probability of γ. Because α is the “strength” of mate preference, larger values of α will reduce the interval width, while the value of δ defines the center of the interval. The value of p is arbitrary, but can be specified as a biologically meaningful value in terms of the impact of the trait difference on the reduction in the mating probability.

## Limitations of the Correlation Coefficient as a Metric of Mate Preference

**Figure 2:**
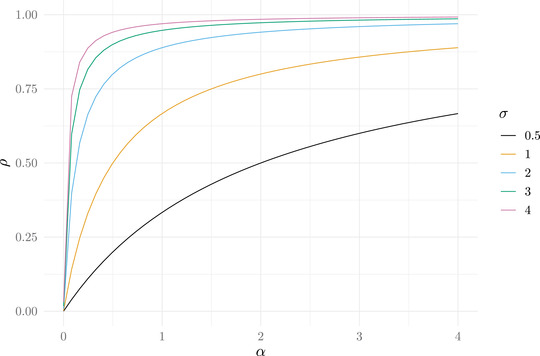
The correlation coefficient (ρ) as a function of mate preference (α) and trait standard deviations ($\sigma_{x}=\sigma_{y}=\sigma$).

The Pearson correlation coefficient is a common metric used by empiricists to infer the existence and strength of preference‐driven assortative mating in a particular population (Jiang et al. 2013) where mate preference is assumed to be the biological process generating a correlation between traits within mated pairs. Even though mate preference does in fact generate a correlation, a sample correlation coefficient cannot be used to estimate the parameter α, which is the strength of mate preference. This is because the correlation of X and Y in mated pairs is a function of both α and the variances of X and Y. To observe the mathematical relationship between a correlation (ρ) and α as a function of a common phenotypic standard deviation, $\sigma_{x}=\sigma_{y}=\sigma$, we numerically computed the correlation between X and Y from the conditional distribution given in equation (3) as a function of α and several fixed values of σ (Fig. 2). Whenever mate preference generates a nonzero correlation between traits within mated pairs, we can see that ρ increases monotonically with respect to α, but the relationship is not linear and varies as a function of σ. More specifically, as α increases, ρ increases faster with larger values of σ until leveling off. As the variance of X−Y increases, more of the distribution of X−Y will fall outside a region of high probability of mating as defined using Figure 1. Overall, for the same given value of α, different values of ρ can be generated depending on the variability of traits in the population, and therefore, a correlation coefficient is not a direct estimate of α and will be misleading if used to infer the strength of mate preference.

## Evaluation of Performance with Simulated Data

To validate our method, we evaluate the performance of the maximum likelihood estimates with simulated data. Males and females composing a breeding population are generated as random samples from normal distributions (except in simulations designed to purposefully violate the normality assumption) with constant means (female: $\mu_{x}=10$; male: $\mu_{y}=12$) and standard deviations (σ=2 for both sexes). Mated pairs are simulated with equation (2) under different fixed values of α and δ, while holding γ=1 constant. Sample sizes $n_{p}$ (number of mated pairs) and $n_{s}$ (total number of single individuals of both sexes, where the number of males is equal to the number of females) vary to demonstrate their influence on the estimates or are fixed at $n_{p}=100$ and $n_{s}=100$. We simulate 1000 replicate datasets for each value of α and δ. Parameter estimates are obtained by maximizing the likelihood function using the R function optim() with the L‐BFGS‐B algorthim (R Core Team 2021).

**Figure 3:**
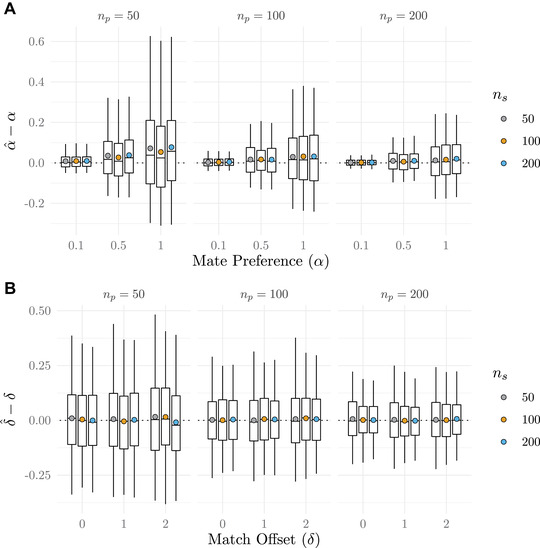
The relationship between true and estimated parameter values under different sample sizes of mated pairs, $n_{p}$, and single individuals, $n_{s}$ (male and female sample size combined). Boxplots show the median, 1st and 3rd quartiles, and 2.5th and 97.5th percentiles, and colored dots show means of the difference in estimates versus true values. Panel A shows results for set values of α, the strength of mate preference, and constant δ=1. Panel B shows results for set values of δ, the match offset, and constant α=0.5.

Figure 3 shows the distance between estimated and true values of α and δ for set values of each parameter under different sample sizes of mated pairs and single individuals. Note that we expect the error around the estimates, $\hat{\alpha }$, to be proportional to the mean of $\hat{\alpha }$ (as seen in Fig. 3A), because α has a lower bound very near zero. This is not the case with $\hat{\delta }$, because δ can be any real number. At larger values of α and smaller sample sizes, there is a tendency for estimated values ($\hat{\alpha }$) to be larger than true values. The bias and error around $\hat{\alpha }$, not surprisingly, decreases with larger samples sizes, particularly when the number of mated pairs is increased. The estimates of δ have little bias, although sample sizes of mated pairs also appear to influence the error surrounding $\hat{\delta }$.

**Figure 4:**
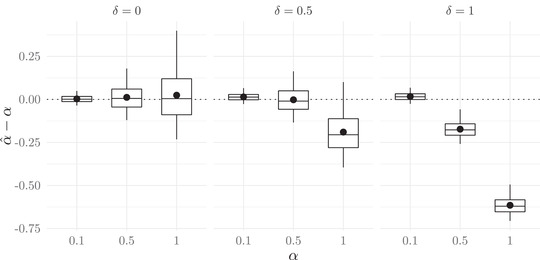
The strength of mate preference, α, is misestimated when the offset parameter, δ, is excluded from the model. This figure shows simulation results comparing true values of α to the model estimates when the parameter δ is not included as a term in equation (2) but is, in fact, nonzero. Boxplots show the median, 1st and 3rd quartiles, and 2.5th and 97.5th percentiles, and black dots show means of the difference in estimates versus true values.

We find that including δ in the model is very important to estimate α without bias whenever the true value of δ is nonzero. If the true value of δ is zero and then not estimated in the model, an accurate estimate of $\hat{\alpha }$ can still be obtained. If the true value of δ is not zero and then not included in the model, $\hat{\alpha }$ becomes less accurate as α and δ increase (Fig. 4). This is because when δ is nonzero, males and females within mated pairs differ from each other, and this difference corresponds to an actual preference. If δ is incorrectly designated as zero, the phenotypic difference between mates then incorrectly estimates a low preference.

**Figure 5:**
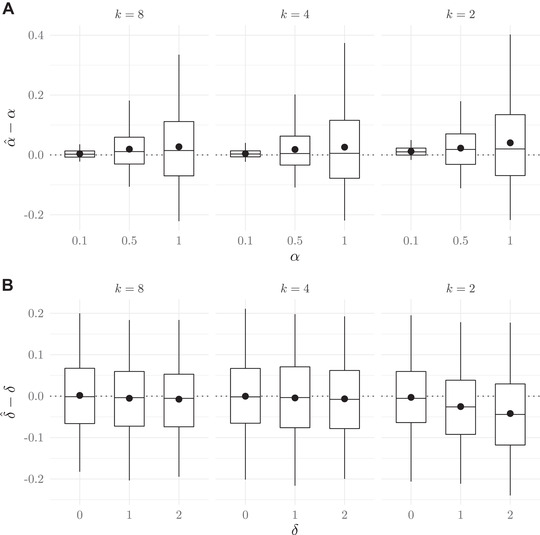
The effect of a skewed phenotypic trait distribution on estimates of α (Panel A) and δ (Panel B). The value of k specifies the shape parameter for generating realizations from a gamma distribution with a scale parameter equal to 4 in all simulations. Boxplots show the median, 1st and 3rd quartiles, and 2.5th and 97.5th percentiles, and black dots show means of the difference in estimates versus true values.

Last, we test the model's performance when we violate two key assumptions: (i) normality and (ii) population homogeneity. Very often quantitative traits are normally distributed or approximately normal, but situations could arise when phenotypic distributions are nonnormal. A probable and problematic example would be a skewed distribution. In Figure 5, we violate the normality assumption by drawing trait values from a gamma distribution with three different shape parameters to simulate skewed distributions of varying degrees. Particularly when the skew is minimal, the parameter estimates for α and δ are robust to this normality violation. We also investigated other examples of nonnormal distributions, specifically heavy‐tailed distributions. These are even less problematic than skewed trait distributions, and therefore the results of these simulations are not presented here.

**Figure 6:**
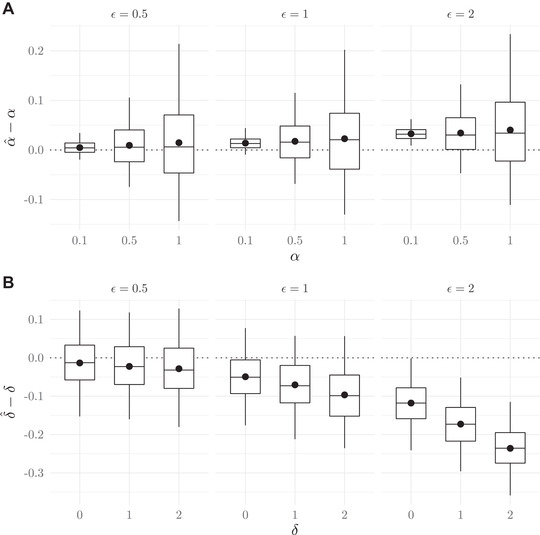
The effect of weak to moderate hidden population structure with phenotypic divergence on estimates of α (Panel A) and δ (Panel B). The value of ε specifies the divergence among phenotypic means for unidentified subpopulations within a single dataset. Boxplots show the median, 1st and 3rd quartiles, and 2.5th and 97.5th percentiles, and black dots show means of the difference in estimates versus true values.

Estimates of the two parameters α and δ can also be biased if mating occurs primarily within unidentified subpopulations, thus violating the assumption that observations are drawn from a homogeneous population. The effect of population structure increases as the subpopulation means get further apart or the variance of each subpopulation distribution decreases. We evaluate the effect of hidden population structure with divergent trait means on the estimates of α and δ with constant sample sizes and a constant population standard deviation (values given in Fig. 6). We simulate three subpopulations with discrete structure (i.e., mating takes place only within each subpopulation), where the value of ε dictates the distance between mean trait values in each subpopulation. For each value of ε (Fig. 6), population means for pairs and single individuals are μ−ε, μ, and μ+ε, respectively. Subsequently, all pairs and single males and females are combined into one dataset to mimic “hidden” population structure. Then, α is estimated as if the observer were unaware of the structure. As expected, the estimate of the strength of mate preference increases as phenotypic divergence among subpopulations increases (Fig. 6A), but the estimates are surprisingly robust, especially at the lower values of ε. The estimate of the offset match is similarly affected by hidden population structure, as the value of $\hat{\delta }$ underestimates the true parameter with increasing values of ε (Fig. 6B). At lower values of ε, this bias is minimal.

## Application to Real Data

Many empirical studies demonstrate the existence of assortative mating by measuring phenotypes and observing mated pairs in the wild. In this section, we apply our likelihood‐based approach to real data from two published studies of size‐assortative mating in three species of marine gastropods from the genus *Echinolittorina* (Ng et al. 2019) and two populations of the European common frog (*Rana temporaria*) (Dittrich et al. 2018). Datasets from both studies are publicly available on Dryad (see Dittrich et al. 2018; Ng et al. 2019). Each study measured body size (shell length in *Echinolittorina* and snout‐vent length (SVL) in *R. temporaria*, both in millimeters), a trait easily observable in males and females in a sample of mated pairs and unmated individuals in each population.

Periwinkles, or marine gastropod molluscs in the family Littorinidae, occur worldwide in the rocky intertidal and have been the subject of numerous evolutionary studies including many studies of sexual selection and mate choice (Ng et al. 2019; Perini et al. 2020). In the genus *Echinolittorina*, courtship is initiated by a male following a female's mucus trail; if this female is deemed an acceptable mate, courtship ends with the male mounting and copulating (Ng et al. 2013). Males are suspected to exhibit size‐dependent mate preference because larger females likely have higher fecundity, but physically copulating with a very large female may not be possible for a smaller male or the risk of sperm competition may be very high and jeopardize paternity if multiple males target the largest females (see Ng et al. 2019).

**Figure 7:**
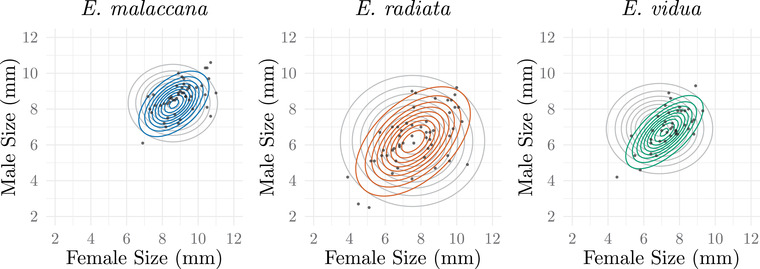
Contour plots show the joint distributions of (X,Y) *mated pairs* under the null expectation of random mating (grey contours) compared to the joint distribution after assortative mating (colored contours) for the three species of *Echinolittorina*. Sample data from shell length measurements (mm) in mated pairs are represented by the scatter plot.

We analyzed data from Ng et al. (2019) using our method. Their study followed individuals throughout the entire mating process in *E. malaccana*, *E. radiata*, and *E. vidua* at Cape d'Aguilar Marine Reserve, Hong Kong in June and July 2012. Our resulting parameter and interval estimates from shell length (millimeters) measurements for the three species of *Echinolittorina* are shown in Table 1. We used likelihood ratio tests to determine if α>0 and δ≠0, which is mate preference with offset matching, significantly differs from α=δ=0, the null expectation of random mating. Results of the likelihood ratio tests are *E. malaccana*: $\chi_{2}^{2}=20.56$, p=0.000034, *E. radiata*: $\chi_{2}^{2}=22.10$, p=0.000016, and *E. vidua*: $\chi_{2}^{2}=31.53$, p=0.00000014 and support the hypothesis that these three species exhibit mate preference with offset matching, albeit to different degrees. To visualize the impact of the estimated values of α and δ on the joint distribution of (X,Y) *mated pairs*, we show contour plots depicting the joint distribution of male and female phenotypes under the null expectation of random mating compared to the estimated distribution under our model with maximum‐likelihood estimates of $\hat{\alpha }$ and $\hat{\delta }$ (Fig. 7). We also calculate the interval $\delta \pm \sqrt{-\log(1-p/100)/\alpha }$ (“Interpreting the Mating Function” section) that defines the range of trait differences (shell length measured in millimeters) where the mating probability is within p=10% of the maximum probability of γ using the estimates for α and δ for each species, *E. malaccana*: (−0.097, 0.92), *E. radiata*: (0.58, 2.20), and *E. vidua*: (0.14, 1.00).

**Table 1:** Parameter and 95% Wald confidence interval estimates from shell length (mm) measurements in three species in the genus *Echinolittorina*

	Species		
Parameter	*E. malaccana*	*E. radiata*	*E. vidua*
α	0.41 ± 0.24	0.16 ± 0.09	0.58 ± 0.31
δ	0.41 ± 0.39	1.39 ± 0.61	0.57 ± 0.35
$\mu_{x}$	8.59 ± 0.18	7.53 ± 0.27	6.88 ± 0.29
$\mu_{y}$	8.33 ± 0.17	6.22 ± 0.31	6.91 ± 0.27
$\sigma_{x}$	1.18 ± 0.13	1.89 ± 0.20	1.40 ± 0.22
$\sigma_{y}$	1.02 ± 0.12	1.71 ± 0.22	1.18 ± 0.02

Similar to the situation in marine gastropods, Dittrich et al. (2018) hypothesize the pattern of assortative mating in *R. temporiana* to be driven by larger males preferring larger and more fecund females. However, under closer investigation this pattern is much more complex and dynamic. Larger individuals of both sexes arrive at breeding sites earlier than smaller individuals, and larger males can out‐compete smaller males for preferred mates. In the face of competition from larger males, the alternative tactic of smaller males is to quickly and indiscriminately mate with any available female, even a small one. Thus, the complex mechanisms leading to size‐assortative mating in *R. temporiana* can generate different outcomes across time and space (Dittrich et al. 2018).

Dittrich et al. (2018) studied two populations of *R. temporaria* from southern and central Germany. The first population, Fabrikschleichach (FS), consisted of a network of 140 ponds where *R. temporaria* typically used between 35 and 40 ponds for reproduction. In contrast, the second population at the locality Kleiwiesen (KW) also contained a network of ponds, but according to the authors, almost the entire population bred within one pond, creating strong male‐male competition for larger female mates. FS population males tended to be smaller than their female mates, whereas the KW population showed the opposite trend, likely due to differences in male densities, in the KW population.

**Figure 8:**
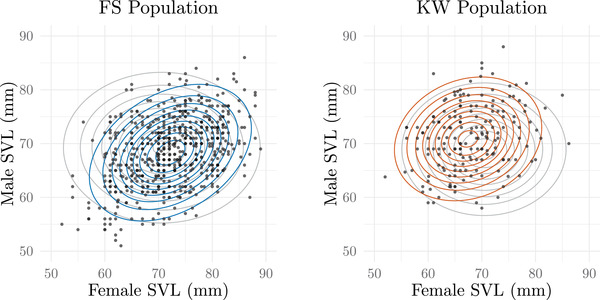
Contour plots show the joint distributions of (X,Y) *mated pairs* under the null expectation of random mating (gray contours) compared to the joint distribution after assortative mating (colored contours) for the two populations of *R. temporaria*. Sample data of SVL measurements (mm) in mated pairs are represented by the scatter plot.

In light of the dynamics of the *R. temporaria* mating system, the authors assessed whether mate preference was adaptive and varied between populations due to mate availability during migration to breeding sites and competition for mates. A summary of the parameter and interval estimates for SVL measurements (millimeters) in each population can be seen in Table 2. Here, after reanalyzing the published dataset in our model, we found no statistical difference in strength of mate preference across the two populations (a 95% Wald confidence interval for the difference in $\alpha_{FS}-\alpha_{KW}$ is 0.0022±0.0024), but similar to the authors' hypothesis, the direction of the offset changed under different conditions (a 95% Wald confidence interval for the difference in $\delta_{FS}-\delta_{KW}$ is 20.21±10.90) (see Appendix for the 95% Wald CI calculation and alternatives to analyzing data from multiple populations). Note that the values of $\hat{\alpha }$ are small. To compensate for numerical instability occurring when α is close to zero, we converted all observations of SVL from millimeters to centimeters. This enlarged the value of $\hat{\alpha }$, and likewise decreased the value of $\hat{\delta }$, ensuring mathematical stability during optimization. These values were converted back to millimeters after the estimates were obtained. Like the analyses in *Echinolittorina*, results of likelihood ratio tests comparing the model of α and δ held constant at zero to the model allowing these parameters to vary are FS: $\chi_{2}^{2}=115.22$, p≈0 and KW: $\chi_{2}^{2}=21.12$, p=0.000026. The strength of mate preference and the difference in the direction of the phenotypic offset between male and female mates can be visualized in Figure 8 for the FS and KW populations. Again, we also calculate the interval $\delta \pm \sqrt{-\log(1-p/100)/\alpha }$ (“Interpreting the Mating Function” section) that defines the range of trait differences (SVL (mm)) where the mating probability is within p=10% of the maximum probability of γ using the estimates for α and δ for each population, FS: (1.38, 10.76) and KW: (−20.40, −7.9).

**Table 2:** Parameter and 95% Wald confidence interval estimates from SVL measurements (mm) for *R. temporaria* in the Fabrikschleichach (FS) and Kleiwiesen (KW) populations

	Population	
Parameter	FS	KW
α	0.0048 ± 0.0011	0.0027 ± 0.0021
δ	6.07 ± 1.62	−14.15 ± 10.78
$\mu_{x}$	70.61 ± 0.94	70.40 ± 2.00
$\mu_{y}$	69.24 ± 0.43	68.95 ± 0.65
$\sigma_{x}$	8.70 ± 0.59	7.20 ± 1.04
$\sigma_{y}$	6.65 ± 0.27	5.81 ± 0.39

## Discussion

A hurdle to testing evolutionary theory surrounding mate preference is the lack of methods allowing theoretical models to interface with observational data (Gavrilets 2014; Servedio et al. 2014). To fill this gap, we have developed a maximum likelihood based approach to estimate parameters in a Gaussian mating function from empirical data. Extensive testing of our model using simulated data demonstrates accurate estimation of the key parameter, the strength of mate preference, α. In addition, we can estimate the optimal offset match in mated pairs, the parameter δ, and the population means and variances in males and females from maximizing a single likelihood function. We discovered that estimating the offset parameter δ is highly necessary to obtain a nonbiased estimate of α, and this parameter also provides valuable information about mate choice mechanisms operating in many mating systems. Best estimation results for both parameters, α and δ, are achieved with larger sample sizes in a well‐mixed population. We also addressed the issue of hidden population structure. Not surprisingly, hidden population structure can skew results, and therefore spatial scale should be carefully considered when using this method (see Rolán‐Alvarez et al. 2015; Estévez et al. 2018). We anticipate empiricists will be aware of strong population structure and be able to use corrected phenotypes (e.g., Langin et al. 2015) in most circumstances, or else we urge consideration of the possibility of hidden structure. If discrete subpopulations can be identified and are believed to share a common α and δ, the likelihood function in equation (A.2) can be used to accommodate structured populations (see Appendix). Even so, parameter estimates appear very robust to undetected weak population structure. As long as the population is mixing so that local distributions of potential mates are similar to the overall population, and this will occur especially as trait variances increase, parameter estimates will have minimal bias and can still be applied to a variety of biological systems.

Applying our method to two published datasets on size‐assortative mating (Dittrich et al. 2018; Ng et al. 2019), we support previous findings and gain additional insight into each mating system. In species of marine gastropods, sexual size dimorphism, with females being the larger sex, is common but not ubiquitous (Ng et al. 2019). However, the mechanism by which males choose mates—preferring females slightly larger than themselves—appears widespread even in species with little to no dimorphism (Saltin et al. 2013; Ng et al. 2019). Ng et al. (2019) investigated the relationship between directional sexual selection on female size and sexual size dimorphism in marine gastropods by studying the mechanisms of male mate choice and patterns of sexual dimorphism in seven different species of marine gastropods. They uncovered a negative correlation between sexual selection intensity on female body size and sexual size dimorphism, and speculate this relationship is caused by the existence of a “similarity‐like” mate choice mechanism (Fernández‐Meirama et al. 2017), where males preferentially mate with females of the same size plus a specific value (Ng et al. 2019). Our likelihood‐based method directly addresses both components of this mate choice mechanism by estimating the parameters α and δ. In our reanalysis of data on three species of *Echinolittorina* included in the Ng et al. (2019) study, we also found strong size‐assortative mating and males preferring larger females respective to their own size, confirming the existence of a “similarity‐like” mate choice mechanism. With parameter estimates in hand, our model could be used to further investigate the relationship between mate preferences generating sexual selection on female size and sexual size dimorphism in these species with simulation.

In the European common frog, mate preferences may be highly labile so that individuals choose mates adaptively. Dittrich et al. (2018) believe multiple mechanisms, such as conditions during migration to breeding ponds and the amount of male‐male competition over mates, all generate the observed patterns of assortative mating and make interpretation difficult. These authors found that the correlation between traits in mated pairs fluctuates across populations. Our reanalysis shows that the strength of mate preference is not statistically different across populations, but the direction of the offset match differs, where males preferred females smaller than themselves in one population, but in the other population, males preferred females larger than themselves. This information is not apparent from the correlations, but is obtained by estimating the parameters separately in our model. Thus, it appears that preference for a particular body size is consistently operating in this species, but local conditions may dictate the optimally sized partner with respect to oneself.

Although our model follows many previous studies that have collectively developed a rigorous theoretical framework to understand mate preference and its evolutionary consequences, we rely on key assumptions that can limit application to all situations. First, we assume phenotypic traits are normally distributed in a homogeneous population. In reality, not all phenotypic trait measurements are independent (e.g., spatial structure would violate this assumption) and normally distributed. Parameter estimates are robust to weak violations of these assumptions as long as the trait is continuous. For example, a skewed distribution such as flowering date (Schmitt 1983; Blionis et al. 2001) can be analyzed with our method, as can populations with weak structure. On the other hand, the model cannot handle categorical phenotypic measurements, which are often important in mating, such as song type in red crossbills (*Loxia curvirostra*) (Snowberg and Benkman 2007), discrete color polymorphisms in strawberry poison frogs (*Oophaga pumilio*) (Yang et al. 2016), alternative male tactics in Trinidadian guppies (*Poecilia reticulata*) (Reynolds et al. 1993), or polymorhic structures in diving beetles (*Graphoderus zonatus*) (Iversen et al. 2019).

Next, we assume that the probability of mating decays in a Gaussian fashion as a function of differences in continuous trait values that can be measured in males and females (eq. 2). This assumption limits the model to matching rules of mate choice and cannot easily be extended to preference/trait rules, unless preference for a particular trait in the opposite sex can be measured as an independent psychological trait on a continuous scale. The preference function we use is unimodal and symmetric, and therefore cannot be ascribed to, for example, a situation where females in a population prefer the largest males. Even in cases where mate choice is made via a matching rule, other types of mating functions that are not unimodal or symmetric, may be better suited to specific systems (Lande 1981; Carvajal‐Rodriguez and Rolán‐Alvarez 2014; Neelon et al. 2019). Models comparable to ours could potentially be built on alternative preference functions, but it may not be possible to write the likelihood function (eq. 6) in closed form as we were able to do here, and numerical approximations would need be used in these cases. Nonetheless, our method also provides the basis for a statistical framework for testing hypotheses about mate preference functions in future studies.

Last, estimation of the strength of mate preference is based on measuring one focal trait. When the data are collected, we assume we have correctly identified and measured this key trait. Mate preference in reality could be very strong but we underestimate it by measuring the wrong trait. Alternatively, we may conclude mate preference is based on the focal trait, but we have merely measured a correlated or indicator trait (Candolin 2003). Measuring an indicator trait may not be a problem if the goal is to understand a process that relies on assortative mating, but this will be misleading if the goal is to unlock key traits involved in mate choice to understand a mating system. Overall, investigators using this method should carefully consider the assumptions in relation to their research objectives, and prior evidence for mate preference based on candidate traits.

Even in the face of limitations, our method has broad implications for the study of mate preference in wild animal and plant populations. Many theoretical models have been constructed to explain phenotypic evolution and divergence that were motivated by observations of mating patterns in nature (Lande 1981). With this foundation firmly in place, it is time for empirical assessment of the role of mate preference in evolutionary dynamics as predicted by models of divergence with gene flow (Dieckmann and Doebeli 1999; Räsänen and Hendry 2008), sexual selection (Servedio and Bürger 2014; Servedio 2016), and the evolution of sexual dimporphism (Bolnick and Doebeli 2003). Our methodology is unique in the use of a likelihood‐based approach to estimate mate preference directly from observational data, thereby allowing an extensive range of systems in which we can study these processes. To help facilitate future studies in this area, we have made our method publicly available and easy to implement in a package called matepref (Clancey and Johnson 2021) for R (R Core Team 2021) that includes functions to estimate the model described in this article, and to simulate data for simulation studies. It is our hope that our method answers previous questions and raises new questions about the role of mate preference in evolutionary dynamics, and does so efficiently to provide a seamless connection between theory and data.

## AUTHOR CONTRIBUTIONS

EC, PAH, and LJH conceived of the project. TRJ and EC developed the statistical model, did the simulations, and analyzed the published datasets. EC wrote the manuscript with input from the coauthors, and TRJ contributed substantially to revisions of the model section. All authors read and approved the final manuscript.

## DATA ARCHIVING

The R code supporting this study can be found at https://github.com/erinclancey/matepref and can be downloaded and implemented as a R package. Datasets from both previously published studies have already been made publicly available on Dryad by the original authors (see Dittrich et al. 2018; Ng et al. 2019).

## CONFLICT OF INTEREST

The authors declare no conflict of interest.

1

Associate Editor: Prof. Michael Kopp

Handling Editor: Dr. Andrew McAdam

## Appendix

This Appendix elaborates on the problem of estimating model parameters across multiple populations. As demonstrated in the Fabrikschleichach (FS) and Kleiwiesen (KW) populations of *R. temporiana*, parameter estimates can be made for each population independently and then compared using interval estimates. A 95% Wald confidence interval for the difference in estimates of α in two populations is

(A.1a)
$$\left(\hat{\alpha_{1}}-\hat{\alpha_{2}}\right)\pm 1.96\times \sqrt{\hat{V}\left(\hat{\alpha_{1}}\right)+\hat{V}\left(\hat{\alpha_{2}}\right)},$$

and a 95% Wald confidence interval for the difference in estimates of δ in two populations is

(A.1b)
$$\left(\hat{\delta_{1}}-\hat{\delta_{2}}\right)\pm 1.96\times \sqrt{\hat{V}\left(\hat{\delta_{1}}\right)+\hat{V}\left(\hat{\delta_{2}}\right)}.$$

Here $\hat{V}$ indicates the estimated variance of each estimator, which is obtained from the diagonal elements of the inverse Hessian matrix after the last iteration of the L‐BFGS‐B algorithm. However, in certain circumstances an investigator may have reason to believe the means and variances across populations are unique, but the populations share a common strength of mate preference, α, and degree of offset match, δ. To make this accommodation, we can expand the likelihood function in equation (6) to accommodate k distinct populations that share α and δ. The likelihood function for multiple populations becomes

(A.2)
$$L\left(\theta \right)=\prod_{k=1}^{K}L_{k}\left(\theta \right),$$

where

$$\begin{array}{ccc} L_{k}\left(\theta \right) & = & \prod_{i\in S_{pk}}\frac{f\left(x_{ik};\mu_{xk},\sigma_{xk}^{2}\right)f\left(y_{ik};\mu_{yk},\sigma_{yk}^{2}\right)e^{-\alpha {\left(x_{ik}-y_{ik}-\delta \right)}^{2}}}{e^{\lambda_{k}t_{k}/\left(1-2t_{k}\right)}/\sqrt{1-2t_{k}}}\\ & & \times \prod_{i\in S_{xk}}f\left(x_{ik};\mu_{xk},\sigma_{xk}^{2}\right)\times \prod_{i\in S_{yk}}f\left(y_{ik};\mu_{yk},\sigma_{yk}^{2}\right),\\ \theta & = & {\left(\alpha ,\delta ,\mu_{x1},\mu_{y1},\sigma_{x1},\sigma_{y1},\ldots ,\mu_{xk},\mu_{yk},\sigma_{xk},\sigma_{yk}\right)}^{\prime },\\ t_{k} & = & -\alpha \left(\sigma_{xk}^{2}+\sigma_{yk}^{2}\right)\;\mathrm{and}\;\lambda_{k}=\frac{{\left(\mu_{xk}-\mu_{yk}-\delta \right)}^{2}}{\left(\sigma_{xk}^{2}+\sigma_{yk}^{2}\right)}. \end{array}$$
